# Fat Graft Transfer in Nasal Tip Reconstruction

**DOI:** 10.1007/s12663-025-02710-1

**Published:** 2025-12-04

**Authors:** Ingrid Raponi, Valeria Pallini, Valentino Valentini, Filippo Giovannetti, Andrea Marzetti

**Affiliations:** 1 ENT and Maxillo Facial Department, Spaziani Hospital, Frosinone, Italy; 2 Maxillo Facial Surgery Department, Sapienza University of Rome, Rome, Italy; 3 Maxillo Facial Surgery Department, University of L’Aquila, L’Aquila, Italy

**Keywords:** Non-melanoma skin cancer, Facial skin defects, Nasal tip reconstruction, Regenerative surgery, Fat graft

## Abstract

**Objective:**

To assess the esthetic and functional outcomes of reconstructing small cutaneous nasal tip defects with fat graft transfer.

**Methods:**

Under local anesthesia, fat graft transfer was performed in nine patients affected by non-melanoma skin cancer of the nasal tip to fill surgical gap. A NAFEQ questionnaire was submitted to all patients to verify nasal function and appearance.

**Results:**

The surgery was uneventful in all cases. In most of the patients (8 of 9 patients), the adipose tissue graft remained vital and re-epithelization started from the edges of the resected area. In three patients, a hematoma in the donor site was observed. All patients were satisfied of esthetical appearance of the nasal tip and of the nasal function.

**Conclusions:**

The fat tissue graft is a good reconstruction option because it gives both good esthetic and functional results, and it can be performed also in elderly patients with many comorbidities.

**Supplementary Information:**

The online version contains supplementary material available at 10.1007/s12663-025-02710-1.

## Introduction

Non-melanoma skin cancer is the most common malignancy in Caucasians, and basal cell carcinoma is the most frequent type [1]

Risk factors for non-melanoma skin cancer are sun exposure, smoking, genetics, most photosensitive skin phototypes, older age, male sex, personal and familiar history of cancer [2, 3]. Most skin carcinomas (80–90%) arise on the sun-exposed parts of the face, and the nose is the most sun exposed [4]. Its reconstruction after surgical removal of the tumor is challenging, as the nose is one of the most difficult facial esthetic units to reconstruct. In addition, facial symmetry and appearance are of utmost importance for self-esteem, personal and social life.

Surgical resection of the lesion in safe margins is the gold standard for the treatment of non-metastatic skin malignancies, and reconstruction of this esthetical subunits should be extremely accurate.

Different approaches have been used to reconstruct nasal skin defects: skin grafts, skin rotation flaps, local flaps (such as the melolabial flap or the forehead flap) and microvascular flaps (i.e., forearm free flap) [5–10]. Since the 1990s, a plethora of new dermal, synthetic, semisynthetic or biological (animal-derived) matrices have been brought to market for advanced full-thickness wound healing [2, 3, 5]. However, the application of synthetic mesh or semisynthetic matrices dressings essentially stimulates the production of granulation tissue and thus may induce fibrosis, causing sometimes an inelastic scar and possible long-term inflammatory side effects [11].

In addition, these biological products have non-negligible costs.

In this article, we propose autologous regenerative medicine, and in particular, fat graft transfers as a cost-effective option to reconstruct cutaneous nasal defects. Our primary objective was to determine duration of complete healing, complication rate and the degree of patient satisfaction.

## Materials and Methods

From December 2020 to December 2022, nine patients affected by non-melanoma skin cancer of the nasal tip were treated.

**Fig. 1:**
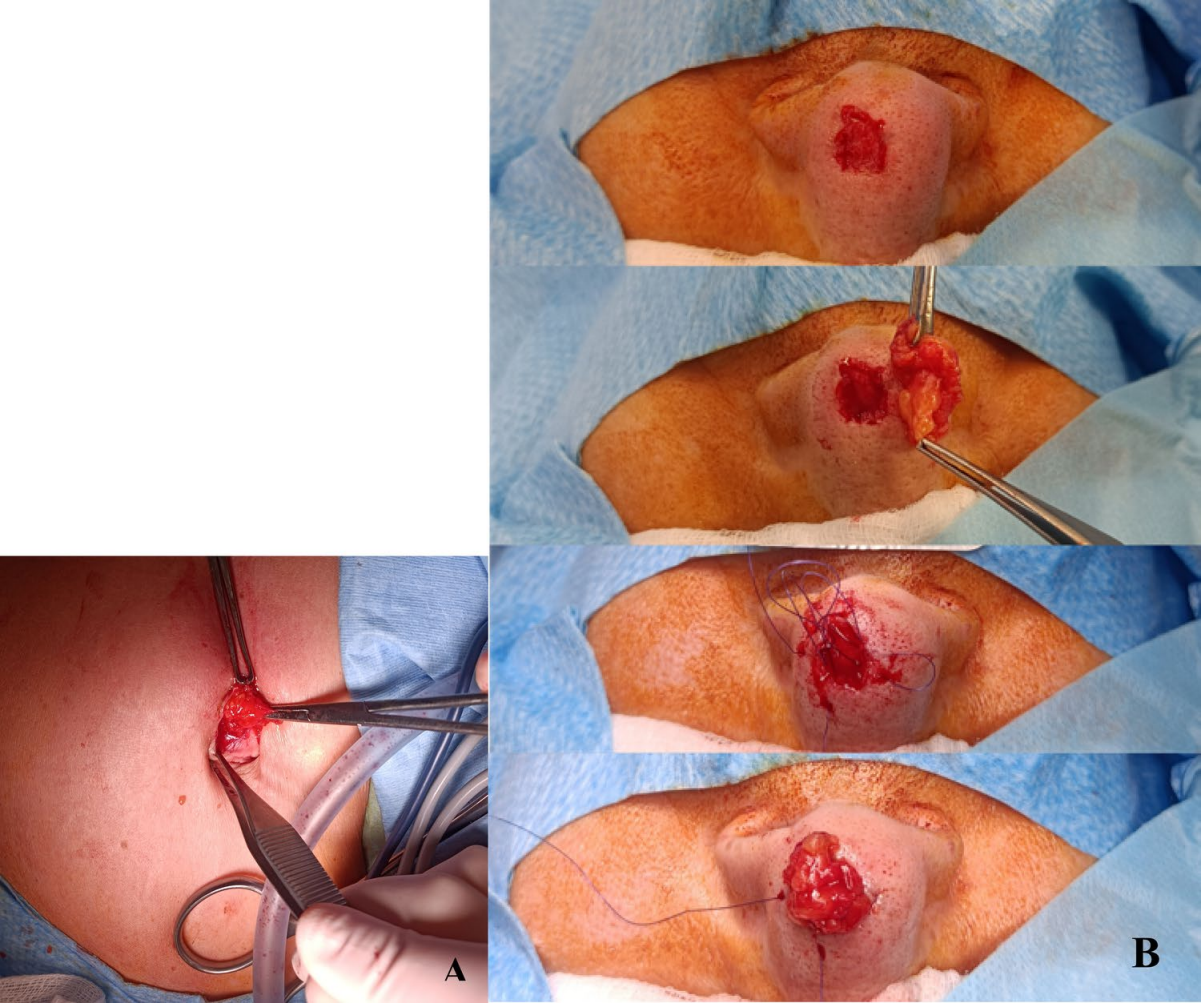
**A** Fat graft harvesting. **B** Fat graft placed and fixed

Under local anesthesia, we performed wide resection of the tumors with a safe margin of 0.3 mm for basal cell carcinoma and 0.5 mm for squamous cell carcinoma. The resection was conducted in depth, up to the cartilaginous plane. The specimen and resection margins were sent for final histopathological examination. Adipose tissue was harvested from a 2-cm paraumbilical incision after infiltration with an anesthetic solution (e.g., lidocaine and ringer lactate) (Fig. 1); to avoid vessel damage due to vasoconstriction, adrenaline was not used. Blunt dissection allows a single round fat graft to be harvested and placed to cover the surgical defect without any kind of washing or centrifugation. The volume of the fat graft taken was deliberately larger than the surgical gap to prevent its resorption (about 30–40%). The fat graft was held in place with Assuplus 4/0 sutures (Assuplus®, Assut Europe S.p.A., Magliano De' Marsi (AQ), Italy) passed from one side to the other of the free edges of the resected area, both for the construction of the ‘mesh’ and for fat fixation (Fig. 1).

Postoperative antibiotic therapy (clavulanic acid and amoxicillin or cephalosporin) was administered for 6 days. Medication consisted in saline solution irrigation, iodine solution disinfection and packing with paraffined gauze, sterile gauze and patch. Postoperative medications were scheduled twice a week for the first two weeks and then once a week for one month. Assuplus Mesh was removed after 8–12 days.

The NAFEQ [12] questionnaire part I (Supp. 1) was submitted to all patients to verify nasal function (score 6 to 30) at the end of re-epithelization process: An overall score higher than 18 was considered good. The NAFEQ [12] questionnaire part II (Supp. 2) was used to verify patients’ satisfaction with nasal appearance (score 8 to 40) at the end of healing process: An overall score higher than 24 was considered satisfying, and a score superior to 32 was considered very satisfying.

## Results

We collected and analyzed retrospectively data of nine patients affected by non-melanoma skin cancer of the nasal tip: Six were female and three were male, and median age was of 72.4 years (Table 1). The mean tumor size was 1.65 × 1.56 cm (with a range of 1.3–2 × 1–1.8). The mean defect size posttumor resection was 1.3 × 1.03 (with a range of 1–1.5 × 0.7–1.3). Surgery was uneventful in all cases, and it took 41.6 min (with a range of 35–50). Patients 1, 7 and 8, who were assuming anticoagulant for cardiologic comorbidities, had an abdominal hematoma restricted to the donor site that resolved in 15 days with glycosaminoglycan polysulphate dermatological gel.

**Table 1:** Clinical and surgical outcomes of Fat Graft Transfer in patients with cutaneous nasal tip defects

Patient	Age	Sex	Diagnosis	Size of defect (cm)	Operative time (min)	Time of complete healing (days)	NAFEQ Part I score	NAFEQ Part II score	Fup (months)	Adverse events
1	72	F	BCC	1.2 × 1.1	35	53	30	39	15	Hematoma of donor site
2	82	F	SCC	1.5 × 1.2	50	40	30	38	24	
3	62	F	BCC	1 × 0.8	40	45	30	40	7	
4	67	F	BCC	1.3 × 0.8	35	42	28	39	4	Partial Lipolysis
5	66	M	BCC	1 × 1.2	45	30	28	40	5	Mild skin depression
6	76	M	BCC	1.2 × 1	50	40	27	40	5	
7	71	F	BCC	1.4 × 1.2	35	33	30	38	4	Hematoma of donor site
8	82	M	BCC	1.5 × 0.7	45	37	28	38	6	Hematoma of donor site
9	84	F	BCC	1.6 × 1.3	40	46	30	40	2	

Supp: supplement; BCC: basocellular carcinoma; SCC: squamocellular carcinoma; Fup: follow-up; Cm: centimeters; Min: minutes

In patient no. 4, the adipose tissue graft underwent partial lipolysis after 20 days, but simultaneously intense granulation and marginal re-epithelization phenomena were observed on the bottom of the surgical wound.

Patient no. 5 during a self-medication inadvertently removed the superficial portion of the fat graft. Fortunately, in the lower part the process of neoangiogenesis had already begun and the healing and re-epithelialization process proceeded normally but a mild depression is visible at 2 months.

**Fig. 2:**
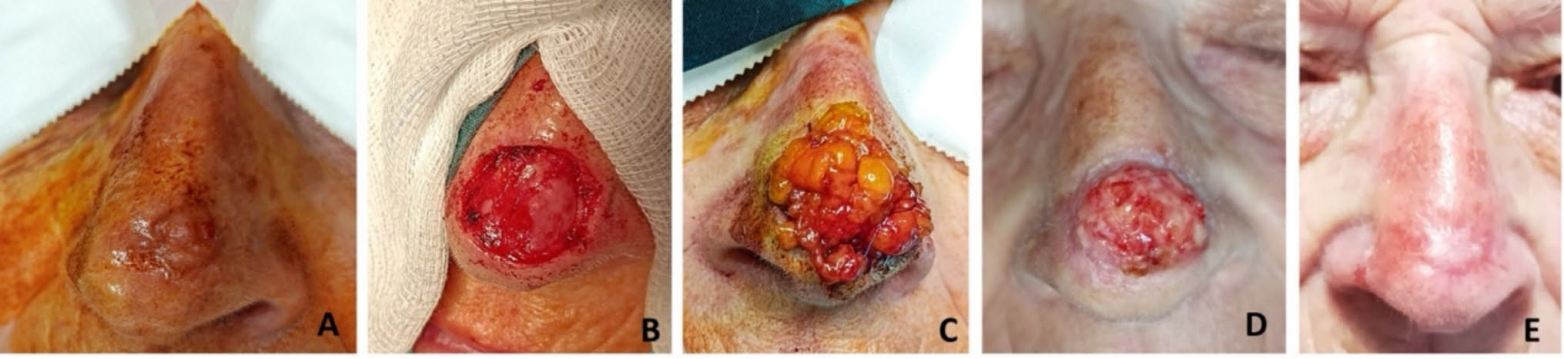
**A** BCC in a 72-year-old female. **B** Tumor resection in wide margins. **C** Fat graft transfer. **D** Postoperative result after 20 days. **E** Postoperative result after 3 months

**Fig. 3:**
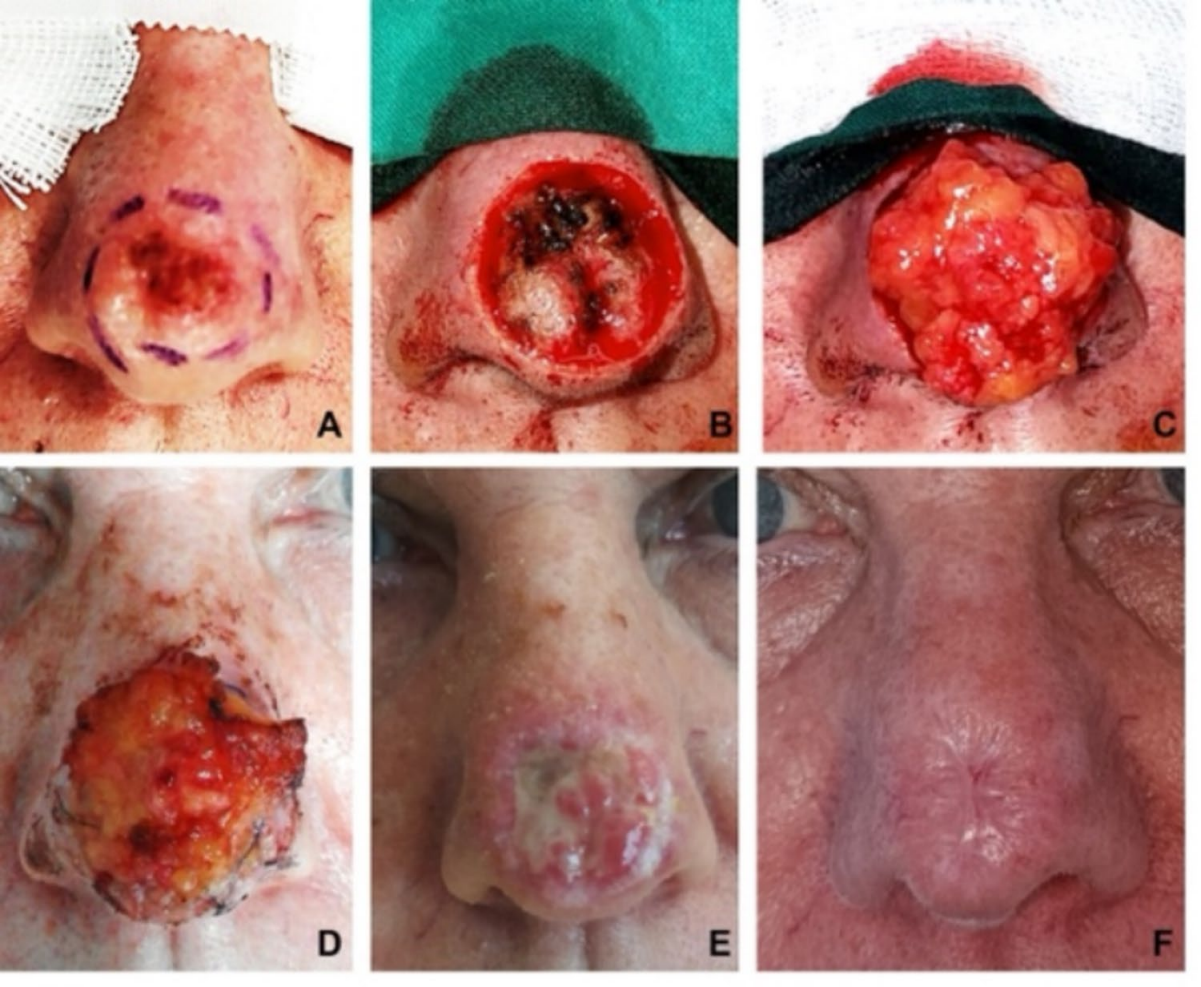
**A** Squamous cell carcinoma in a 82 years old female; **B** wide resection of the tumor; **C** fat graft transfer; **D** postoperative outcome after 7 days; **E** postoperative outcome after 20 days; **F** postoperative outcome after 45 days

In remaining patients, the adipose tissue graft remained vital and re-epithelization started from the edges of the resected area (Figs. 2, 3).

A complete healing of the surgical site was observed after 40.6 days (with a range of 30–53 days) (Figs. 2, 3). The use of a non-absorbable polyether polyurethane monofilament suture with its characteristic’s excellent compliance of elasticity, smoothness and absence of capillarity gives an added value to the technique with its benefits for the patients.

Thereafter, patients were advised to apply total sunscreen and creams with high hyaluronic acid content.

According to NAFEQ part I questionnaire, no patient complained of respiratory distress after surgery: A mean score of 29 (with a range of 27–30) was obtained in NAFEQ part I questionnaire. In NAFEQ part II questionnaire, a mean score of 39.1 (with a range of 38–40) was obtained, and patients were satisfied with the esthetic outcome (Table 1).

## Discussion

Many reconstructive options can be performed depending on skin defect wideness: full-thickness skin grafts, composite grafts like perichondral cutaneous composite grafts or skin/cartilage composite grafts, local or regional flaps [13]. All these techniques, however, are not free from complications and require perioperative and postoperative cares. Moreover, these techniques are associated with multiple scars that, although they may follow discharge lines that make them less visible, are still long and located in the center of the face.

Large defects of the nasal tip require local flaps that can sometimes take more than an hour, causing discomfort for patients, especially the elderly, while regional flaps are usually performed under general anesthesia, so not all patients can undergo major surgery. In this regard, compared with dermal matrices, fat grafting allows the skin and subcutaneous defect to be restored while containing costs.

Another common problem is trap door deformity: Circular scar contracts during wound healing causing elevation of the flap that results in a pin-cushion appearance that often needs a second surgery [9].

Our free fat grafting is a good reconstruction option because it takes advantage of re-epithelialization and natural wound healing. The fat supports the tissue, generating skin that is entirely like the surrounding skin, with no color mismatch or tissue excessive depressions. This reconstructive technique has been employed by the authors in the management of scalp defects, yielding highly satisfactory results [14]. Surgery can be performed under local anesthesia also in elderly patients and those patients with comorbidities or anticoagulant therapies that do not need to be suspended. Our experience also showed that although the fat is uncovered, no infection was observed in any of the patients, even in those with diabetes and other comorbidities. Fat necrosis/lipolysis is also an infrequent event and usually involves the outermost, superficial portion. Finally, the proposed technique has some shortcomings including the long healing time, the common formation of hematoma at the donor site, the need for numerous dressings, and some cautions to achieve optimal esthetic results. Regarding this last point, we found no difficulties in properly instructing patients, even those who seemed less compliant, and likewise, limited to our experience, patients did not complain about the numerous dressings, or the costs associated with them. Regarding the first point, it must be considered that reconstruction with a forehead flap requires a second surgical time for flap autonomization and is therefore associated with a total healing time quite similar to that of fat grafting with a greater discomfort for the patient of having a bulky dressing that occupies the entire central part of the face for many days.

For these reasons, we believe that fat graft is a good reconstructive option in nose reconstruction after full-thickness excision of malignancies.

To our knowledge, this is the first case series in which autologous adipose tissue grafting was used to repair a full-thickness skin defect of the nasal tip.

## Conclusions

According to our experience, autologous adipose tissue grafting gives a satisfactory esthetic result, and it is a simple and reproducible procedure that takes less than 50 min, which can be used in elderly or complicated patients since it can be carried out under local anesthesia. It could be the desirable choice for those patients who are scared from surgery or who does not want to undergo to major procedures. These features make autologous adipose tissue grafting a valid alternative to local or loco-regional flaps, which require adjunctive and extensive skin scares difficult to hide and prolonged general anesthesia which frequently makes elderly patients or patients with comorbidities not eligible.

## Supplementary Information

Below is the link to the electronic supplementary material.

Supplementary file1 (DOCX 13 kb)

## Data Availability

All data generated or analyzed during this study are included in this published article.
