# Supplementary material for: Fat Graft Transfer in Nasal Tip Reconstruction
Source: J Maxillofac Oral Surg. 2025 Dec 4;25(4):1018–22. doi: 10.1007/s12663-025-02710-1 (PMC13415733; doi:10.1007/s12663-025-02710-1)
Supplement: Supplementary file 1 — Supplementary file1 (DOCX 13 kb) [file 12663_2025_2710_MOESM1_ESM.docx]

**Supplement 1**

|  | **Always** | **Mostly** | **Every now and then** | **Hardly ever** | **Never** |
| --- | --- | --- | --- | --- | --- |
| How often do you have trouble breathing through your nose? | 1 | 2 | 3 | 4 | 5 |
| How often do you snore? | 1 | 2 | 3 | 4 | 5 |
| How often can you smell odors? | 1 | 2 | 3 | 4 | 5 |
| How often do you have trouble with nasal crusts? | 1 | 2 | 3 | 4 | 5 |
| How often do you have a bloody nose? | 1 | 2 | 3 | 4 | 5 |
|  | **Very poor** | **Poor** | **Moderately** | **Good** | **Excellent** |
| How do you assess your quality of speech? | 1 | 2 | 3 | 4 | 5 |

**Supplement 2**

|  | **Very dissatisfied** | **Dissatisfied** | **Moderately** | **Satisfied** | **Very satisfied** |
| --- | --- | --- | --- | --- | --- |
| How satisfied are you with your total nasal functioning? | 1 | 2 | 3 | 4 | 5 |
| How satisfied are you with your nasal tip appearance? | 1 | 2 | 3 | 4 | 5 |
| How satisfied are you with your nasal wing(s) appearance | 1 | 2 | 3 | 4 | 5 |
| How satisfied are you with your nasal dorsum appearance? | 1 | 2 | 3 | 4 | 5 |
| How satisfied are you with the size of your nostril(s)? | 1 | 2 | 3 | 4 | 5 |
| How satisfied are you with the color of your nasal skin? | 1 | 2 | 3 | 4 | 5 |
| How satisfied are you with your nasal position? | 1 | 2 | 3 | 4 | 5 |
| How satisfied are you with your total nasal appearance? | 1 | 2 | 3 | 4 | 5 |
